# Confocal laser scanning microscopy analysis of *S. epidermidis* biofilms exposed to farnesol, vancomycin and rifampicin

**DOI:** 10.1186/1756-0500-5-244

**Published:** 2012-05-16

**Authors:** Nuno Cerca, Fernanda Gomes, Sofia Pereira, Pilar Teixeira, Rosário Oliveira

**Affiliations:** 1 IBB-Institute for Biotechnology and Bioengineering, Centre of Biological Engineering, University of Minho, Campus de Gualtar, Braga, 4710-057, Portugal

## Abstract

**Background:**

*Staphylococcus epidermidis* is the major bacterial species found in biofilm-related infections on indwelling medical devices. Microbial biofilms are communities of bacteria adhered to a surface and surrounded by an extracellular polymeric matrix. Biofilms have been associated with increased antibiotic tolerance to the immune system. This increased resistance to conventional antibiotic therapy has lead to the search for new antimicrobial therapeutical agents. Farnesol, a quorum-sensing molecule in *Candida albicans*, has been described as impairing growth of several different microorganisms and we have previously shown its potential as an adjuvant in antimicrobial therapy against *S. epidermidis.* However, its mechanism of action in *S. epidermidis* is not fully known. In this work we better elucidate the role of farnesol against *S: epidermidis* biofilms using confocal laser scanning microscopy (CLSM).

**Findings:**

24 h biofilms were exposed to farnesol, vancomycin or rifampicin and were analysed by CLSM, after stained with a Live/Dead stain, a known indicator of cell viability, related with cell membrane integrity. Biofilms were also disrupted by sonication and viable and cultivable cells were quantified by colony forming units (CFU) plating. Farnesol showed a similar effect as vancomycin, both causing little reduction of cell viability but at the same time inducing significant changes in the biofilm structure. On the other hand, rifampicin showed a distinct action in *S. epidermidis* biofilms, by killing a significant proportion of biofilm bacteria.

**Conclusions:**

While farnesol is not very efficient at killing biofilm bacteria, it damages cell membrane, as determined by the live/dead staining, in a similar way as vancomycin. Furthermore, farnesol might induce biofilm detachment, as determined by the reduced biofilm biomass, which can partially explain the previous findings regarding its role as a possible chemotherapy adjuvant.

## Findings

### Background

*Staphylococcus epidermidis*, a normal inhabitant of human skin and mucosa, has recently emerged as a leading cause of biofilm-related infections, particularly, in patients with indwelling medical devices [1,2] due to its ability to adhere to abiotic surfaces and to form biofilms [2,3]. Biofilms are often defined as three-dimensional communities of microorganisms that are attached to a surface and encased in an extracellular matrix composed mainly of polysaccharides, proteins and extracellular DNA [4,5]. The major virulence factor of *S. epidermidis* is biofilm formation [6] and cells in biofilms are normally more tolerant to antibiotics than planktonic cells [7-9], making drug resistance in a *S. epidermidis* biofilm-related infection a serious problem, especially in nosocomial infections [10]. *S. epidermidis* are also inherently resistant to host defense mechanisms [11,12]. The increased resistance to conventional antibiotic therapy led to the search for new therapeutical agents.

Despite concerted efforts to treat biofilm-related infections with antibiotic therapy, the physical removal of the infected medical device is often necessary [13], which carries an additional economic and health cost. Therefore, novel strategies are necessary to combat biofilm-related infections to improve clinical outcomes.

Farnesol is a sesquiterpene alcohol and is the first quorum-sensing molecule described in eukaryotes [14] and produced by most *Candida* species [15]. Farnesol has been shown to have antimicrobial activity in many species [16], including the coagulase positive *Staphylococcus aureus*[17,18] and the coagulase negative *Staphyloccous epidermidis*[19]. Recently, we showed that farnesol could act as an adjuvant against *S. epidermidis* infections, when combined with common antibiotics such as vancomycin, tetracycline and rifampicin. However, this effect was more proeminent in planktonic cells and was significantly reduced in biofilms [20]. Nevertheless, Pammi *et al*. had shown that combinations of farnesol with other antibiotics were also effective in reducing *S. epidermidis* biofilm biomass [21].

Despite the promising role of farnesol as an adjuvant in antimicrobial activity against *S. epidermidis*, little is known regarding its mechanism of action. We have previously shown that the biofilm matrix composition and distribution of *S. epidermidis* biofilms was affected by farnesol [22]. In this study we present a more in-depth analysis of biofilm structure, viability and biomass changes when in contact with farnesol, and compare it with biofilms exposed to vancomycin or rifampicin.

## Material and methods

### Bacterial strains and growth conditions

The biofilm-producing strain used in this study, *S. epidermidis* 9142,was previously described [23]. This strain was originally isolated from blood cultures from an infected central venous catheter by Mack *et al*. [24], Strains were grown as previously described, in Tryptic Soy Broth (TSB) and Tryptic Soy Agar (TSA) (Oxoid, Cambridge, UK) [25]. Briefly the strain was inoculated into 15 mL of TSB from TSA plates not older than 2 days and grown for 18 (± 2) h at 37°C in an orbital shaker at 130 rpm. Cells were harvested by centrifugation (for 10 min at 9500 × g and 4°C), and resuspended in TSB supplemented with 0.25% glucose (TSBG) (Fisher Scientific, Waltham, MA, US) adjusted to an optical density (640 nm) equivalent to 1 × 10^9^ cells mL^-1^ and then used in the subsequent assays.

### Biofilm challenge with farnesol, vancomycin or rifampicin

Biofilm challenge was done as described before [26], with some modifications. Briefly, biofilms were formed during 24 h at 37°C and 120 rpm on 96 (for killing assays) or 6-well tissue culture (for confocal analysis) plates by dispensing 4 mL of a 1 × 10^9^ cells mL^-1^ cell suspension in TSBG. Then, the growth medium was removed and replaced with fresh TSBG supplemented with either 300 μM of farnesol (Sigma, St Louis, MO, US) [19], or the antibiotics at the respective peak serum concentration (vancomycin: 40 mg/L and rifampicin: 10 mg/L) (Sigma, St Louis, MO, US) [9]. Biofilms were exposed to the respective antimicrobial agents for 2, 4, 6, 8 or 24 h. After exposure, biofilms were sonicated for 10 seconds at 10 W (Ultrasonic Processor, Cole-Parmer, USA). This procedure did not reduced cell viability, as showed before [9]. The cell suspension was washed twice to prevent antibiotic carry over and resuspended in 0.9% NaCl. Viable bacteria were determined by standard colony-forming-units (CFU) plating in TSA plates. This experiment was repeated three times with duplicates.

### Confocal scanning laser microscopy (CLSM) analysis of the biofilms

CSLM was performed as described before [22]. Briefly, 24 h after the biofilms were exposed to farnesol, vancomycin or rifampicin, they were washed twice with 0.9% NaCl. Biofilm cell viability was determined with live/dead staining (Molecular Probes, USA) following the manufacturer´s instructions. A negative control was used to determine the baseline threshold for dead cells, by killing the biofilm with 96% ethanol for 4 h. The plates were incubated for 20 min at room temperature in the dark. After staining, the biofilms were gently rinsed with 0.9% NaCl. The biofilm images were acquired in an Olympus^TM^ FluoView FV1000 (Olympus, Lisboa, Portugal) confocal scanning laser microscope. Biofilms were observed using a 60x water-immersion objective (60x/1.2 W). For each condition, three independent biofilms were used. Images were acquired with 512 x 512 resolutions in at least four different regions of each surface analyzed. For biofilm maximum thickness determination, at least twenty different regions per surface were analyzed, by determining the the top and bottom layer of the biofilm, and calculating the maximum thickness of each region.

### Statistical analysis

Quantitative assays were compared using unpaired *t*-test or one-way analysis of variance (ANOVA) by applying Levene’s test of homogeneity of variances and the Tukey multiple comparisons test. All tests were performed with a confidence level of 95%.

## Results and discussion

Antibiotic resistance is a serious problem in *S. epidermidis* since many clinical isolates of this organism are resistant up to eight different antibiotics [10]. Farnesol is one promising candidate to be used as an adjuvant in antimicrobial chemotherapy against biofilms. Some studies have indicated a possible interaction of farnesol with cell membranes of *S. aureus*, resulting in a reduced cell membrane integrity [18]. We have recently shown that farnesol increased cell death by the action of tetracycline or rifampicin [20], possibly due to the permeabilization of the cell wall [22]. The same was true in combination with *N*-acetylcysteine [27]. However, the exact mechanism of farnesol action in *S. epidermidis* is not fully known. A recent study of Zhu *et al.*[28] revealed that farnesol induced *Candida albicans* apoptosis by conjugating with intracellular glutathione, inducing a strong oxidative stress and eventual cell death. However, as *C. albicans* biology is fundamentally different from *S. epidermidis*, it is not possible to infer a similar role of farnesol in *S. epidermidis* biofilms.

**Figure 1:**
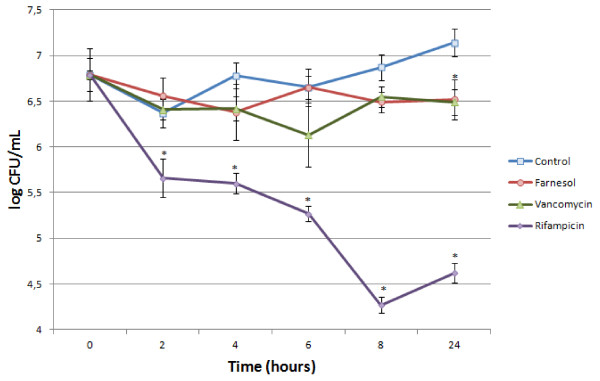
Effect of farnesol, vancomycin, and rifampicin on viability of S. epidermidis biofilm cells versus time of treatment.* statistically different as compared with the control, at the respective time-point (p < 0.05).

Based on our previous findings [19,20,22,27], we selected two antibiotics with distinct mechanisms of action: vancomycin, that inhibits the cell wall synthesis and shows low activity against *S. epidermidis* biofilms, and rifampicin, a RNA synthesis inhibitor, that shows high activity against *S. epidermidis* biofilms and compared their effects with farnesol action on biofilm structure and cell viability. Figure 1 shows a time-course study on viable cells after exposure to the three antimicrobial agents. As expected, vancomycin was not very effective in killing biofilm bacteria. In fact, as compared with the control, the only significant difference found was at 24 h of exposure. On the other hand, rifampicin was able to kill more than 1 log of bacteria in the first two hours of exposure, and more than 2.5 log after 8 h. Farnesol showed similar results as vancomycin in all time periods studied. 

**Figure 2:**
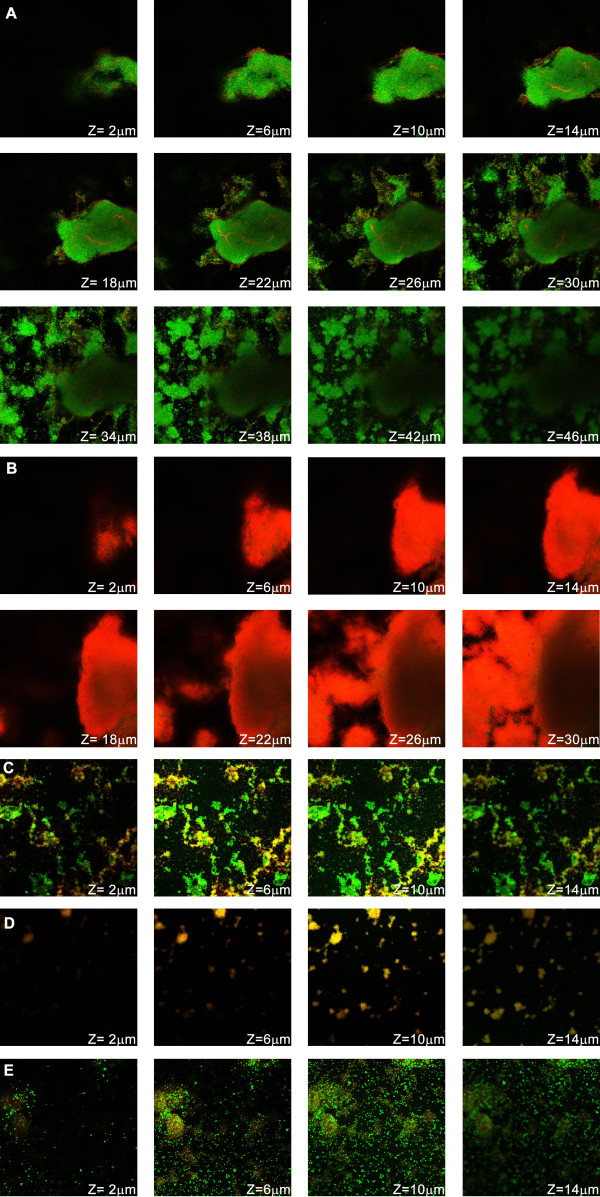
**Effect of farnesol and antibiotics on biofilm structure and viability.(A)** Biofilm without antimicrobial agent; **(B)** Negative control for live/dead staining, treated with 96% ethanol for 4 h; **(C)** Biofilm exposed to 300 μM farnesol for 24 h; **(D)** Biofilm exposed to 40 mg/L of vancomycin for 24 h; (**E**) Biofilm exposed to 10 mg/L of rifampicin for 24 h.

**Figure 3:**
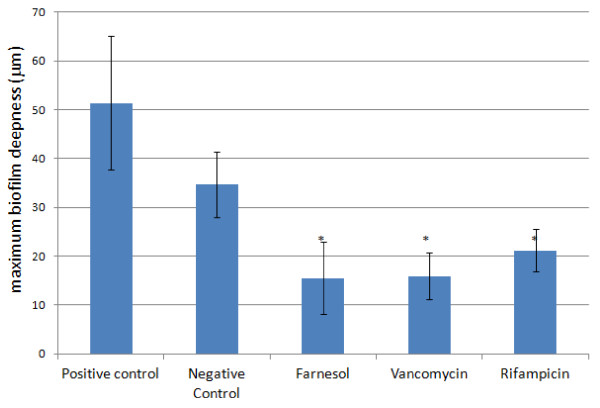
**Effect of farnesol and antibiotics on biofilms maximum depth.** * statistically different as compared with the control (p < 0.05).

CLSM has been widely used to study biofilm structure, composition and metabolism in several different microorganisms [29-31]. One of the big advantages of CLSM is that it allows in-depth analysis of biological structures, without killing or damaging the biological structure [32]. Live/dead staining has been used as indicator of cell viability, as determined by the integrity of the cell wall membrane in many bacterial populations, including biofilms [19,33]. When we analyzed the biofilm structure and viability by CLSM an interesting result was found: despite the fact that vancomycin and farnesol were not able to effectively kill biofilm bacteria, biofilm biomass was strongly reduced, as can be seen qualitatively in Figure 2 or quantitatively in Figure 3. In fact, the biofilm maximum depth, an indirect measure of the amount of biofilm biomass was equally reduced when treated with any of the three different antimicrobial compounds used (Figure 3). For better comparison of the antimicrobials action, regions of biofilms with similar deepness were chosen (Figure 2C, D, E). More images can be found in supplementary information (Additional file 1, Additional file 2, Additional file 3 and Additional file 4). It is well known that bacterial biofilms are heterogeneous communities, regarding both the three dimensional structure but also the physiology of in vitro biofilm bacteria [4]. It was recently described that *S. epidermidis* biofilms have a variable sub-population of cells presenting a partially damaged cell membrane. This sub-population was able to incorporate propidium iodide at low levels, but were still viable bacteria [34]. In the present study, biofilms treated with vancomycin or farnesol showed clusters of live (green) and death bacteria (red) but also a sub-population of bacteria with somewhat damaged cell membrane (yellow). Despite the fact that biofilms exposed to vancomycin or farnesol had smaller amounts of biomass, the typical *S. epidermidis* biofilm high density cell clusters were still detected [35-38]. On the other hand, rifampicin-treated biofilms hardly presented regions of live or somewhat damage cell clusters. While live and viable bacteria were mainly dispersed in small microcolonies, the few cluster found were of dead bacteria (Figure 2E). 

Since it is well known that biofilm cells are metabolically less active than planktonic ones [39] and some cells are in a dormant state [40], antibiotics that require active cell division, such as vancomycin, are highly hindered in such conditions. Nevertheless, the reduced biomass present suggested that both agents targeting the cell wall were able to, somehow, induce cell detachment. While *S. epidermidis* biofilms have been proposed to partially detach fragments of the biofilm [6] in order to potentially colonize other regions of the host, cell detachment is not yet fully understood, however some genetic regulation might be involved [41]. Recently Kaneko et al. showed that farnesol interfered with the *S. aureus* mevalonate pathway, which is related with cell membrane maintenance and protein anchoring, among other important cell functions [42]. *S. aureus* biofilm production and metabolism is similar to *S. epidermidis*[43]. It has been shown before that the lack of proper protein anchoring results in reduced biofilm formation in *S. aureus* and *S. epidermidis*, related with regulation of poly-N-acetyl-β-(1–6)-glucosamine deacetylation [11,44].

## Conclusions

Treatment of *S. epidermidis* biofilms with farnesol and vancomycin did not significantly decrease cell viability. However, they both appeared to reduce the biomass in these biofilms. Our previous reports regarding the role of farnesol as an adjuvant in antimicrobial chemotherapy can now be better explained, since released cells from the biofilms would potentially be more prone to antibiotic attack, taking in consideration that no diffusion barrier would be present in such cases [45]. Kaneko *et al*. [42] results and ours further provide more evidence that farnesol mechanism of action in *S. epidermidis* is related with the cell wall membrane integrity. We are currently exploring the role of farnesol in *S. epidermidis* cell membrane integrity as well as its impact on biofilm physiology.

## Competing interests

The authors declare that they have no competing interests.

## Authors contributions

NC did the confocal microscopy analysis and writing of the manuscript. FG and PT did the timelapse studies. SP and FG prepared the samples for confocal analysis. NC and RO planned the experiments. All authors read and approved the final manuscript.

## Supplementary Material

Additional file 1 **Figure S1.**Other examples of biofilms exposed with rifampicin. Click here for file

Additional file 2 **Figure S2.**Other examples of biofilms exposed with vancomycin. Click here for file

Additional file 3 **Figure S3.**Other examples of biofilms exposed with (1), (2), (3) farnesol, (4) or the controls. Click here for file

Additional file 4 **Figure S4.**Other examples of biofilms not exposed to antimicrobial agents. Click here for file
