# Supplementary figures and images for: Confocal laser scanning microscopy analysis of S. epidermidis biofilms exposed to farnesol, vancomycin and rifampicin
Source: BMC Res Notes. 2012 May 16;5:244. doi: 10.1186/1756-0500-5-244 (PMC3481475; doi:10.1186/1756-0500-5-244)

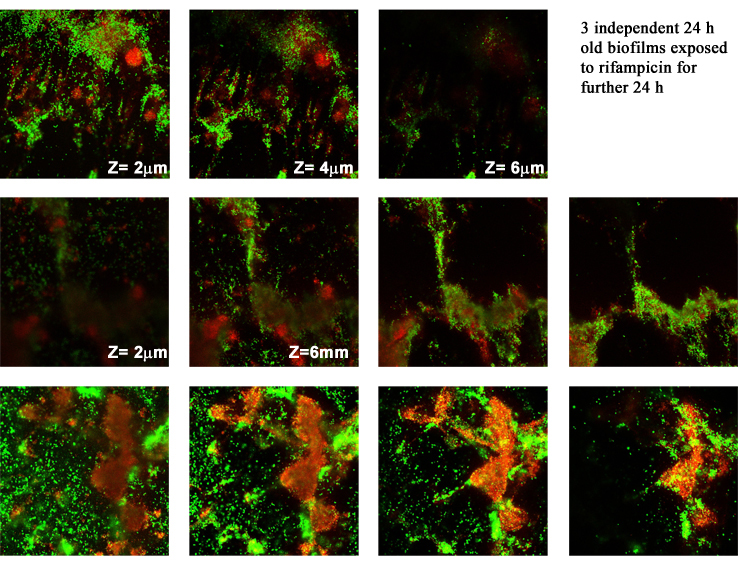

Supplement: Additional file 1 — Figure S1.Other examples of biofilms exposed with rifampicin. [file 1756-0500-5-244-S1.jpeg]

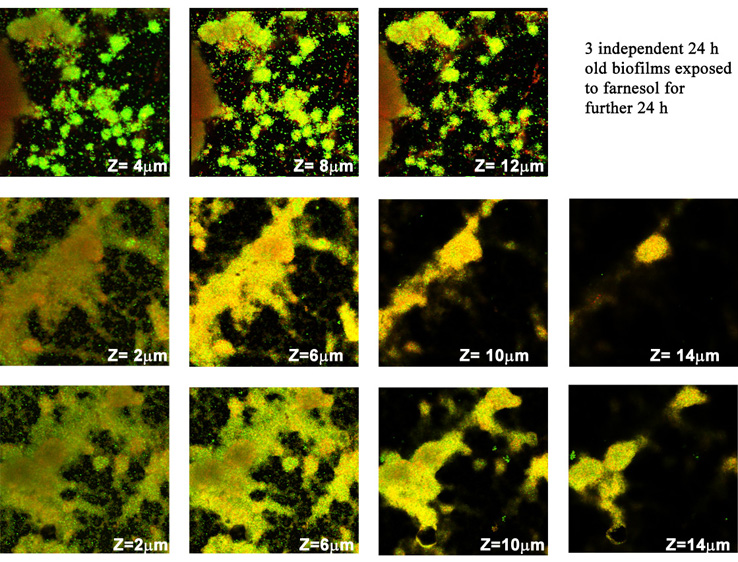

Supplement: Additional file 2 — Figure S2.Other examples of biofilms exposed with vancomycin. [file 1756-0500-5-244-S2.jpeg]

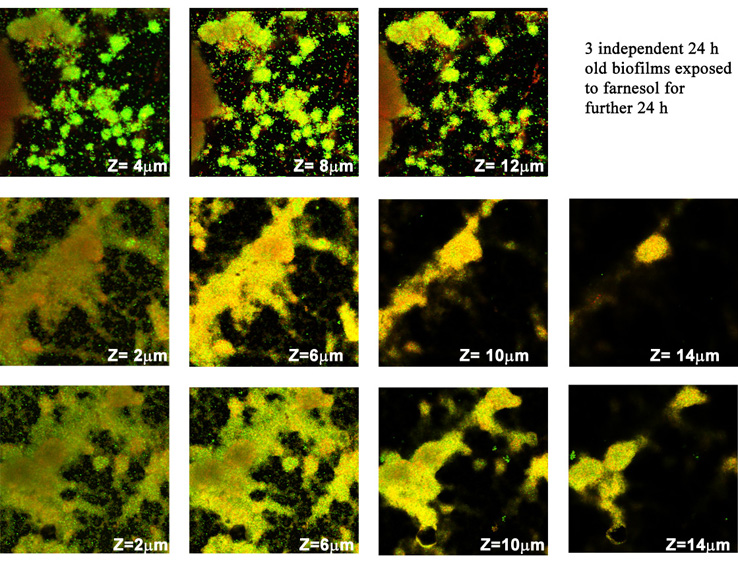

Supplement: Additional file 3 — Figure S3.Other examples of biofilms exposed with (1), (2), (3) farnesol, (4) or the controls. [file 1756-0500-5-244-S3.jpeg]

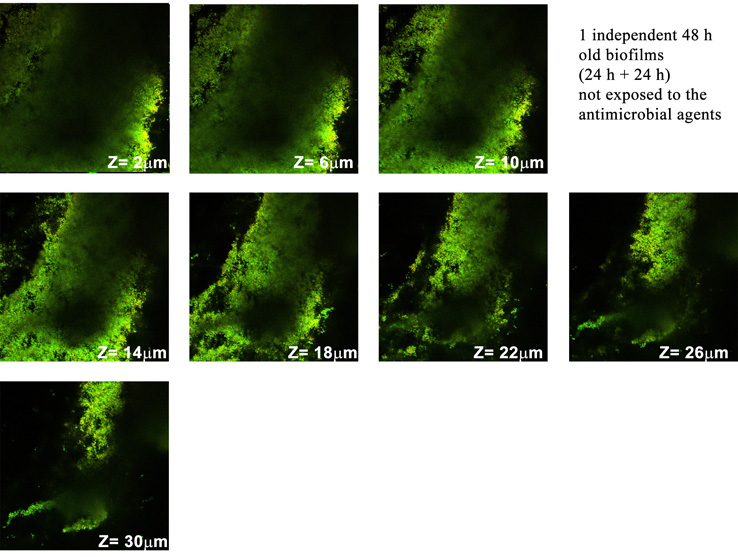

Supplement: Additional file 4 — Figure S4.Other examples of biofilms not exposed to antimicrobial agents. [file 1756-0500-5-244-S4.jpeg]
